# LTCC and Bulk Zn_4_B_6_O_13_–Zn_2_SiO_4_ Composites for Submillimeter Wave Applications

**DOI:** 10.3390/ma14041014

**Published:** 2021-02-21

**Authors:** Dorota Szwagierczak, Beata Synkiewicz-Musialska, Jan Kulawik, Norbert Pałka

**Affiliations:** 1 Łukasiewicz Research Network—Institute of Microelectronics and Photonics, Kraków Division, ul. Zabłocie 39, 30-701 Kraków, Poland; beata.synkiewicz.musialska@imif.lukasiewicz.gov.pl (B.S.-M.); jan.kulawik@imif.lukasiewicz.gov.pl (J.K.); 2 Institute of Optoelectronics, Military University of Technology, ul. gen. W. Urbanowicza 2, 00-908 Warszawa, Poland; norbert.palka@wat.edu.pl

**Keywords:** Zn_4_B_6_O_13_-Zn_2_SiO_4_ composite, low temperature cofired ceramics, dielectric properties, THz time domain spectroscopy, submillimeter wave applications

## Abstract

New zinc metaborate Zn_4_B_6_O_13_–willemite Zn_2_SiO_4_ composites were investigated as promising materials for LTCC (low temperature cofired ceramics) substrates of microelectronic circuits for submillimeter wave applications. Composites were prepared as bulk ceramics and LTCC multilayer structures with cofired conductive thick films. The phase composition, crystal structure, microstructure, sintering behavior, and dielectric properties were studied as a function of willemite content (0, 10, 13, 15, 20, 40, 50, 60, 100 wt %). The dielectric properties characterization performed by THz time domain spectroscopy proved the applicability of the composites at very high frequencies. For the 87% Zn_4_B_6_O_13_–13% Zn_2_SiO_4_ composite, the best characteristics were obtained, which are suitable for LTCC submillimeter wave applications. These were a low sintering temperature of 930 °C, compatibility with Ag-based conductors, a low dielectric constant (5.8 at 0.15–1.1 THz), a low dissipation factor (0.006 at 1 THz), and weak frequency and temperature dependences of dielectric constant.

## 1. Introduction

The development of modern wireless communication systems (5G, 6G) has created a permanent demand for new dielectric substrate materials to fulfill the needs of the increased signal transmission rate, enhanced miniaturization, and improved possibility of integrating passive elements [1,2,3,4]. The requirements for such new materials include a low dielectric constant to minimize the signal propagation delay, a low dielectric loss to ensure frequency selectivity and to restrict power consumption, and a low sintering temperature to enable the use of multilayer LTCC/ULTCC (low/ultralow temperature cofired ceramics) technology. Along with the modification of materials with a low dielectric constant, such as silica, borosilicate glasses, cordierite, mullite, forsterite, diopside, willemite, and aluminates [5,6,7,8,9,10,11,12,13,14,15,16,17,18,19,20,21,22,23,24], which have been well-known for decades, less popular ceramics have been explored recently, such as borates, tungstates, molybdates, vanadates, and phosphates [25,26,27,28,29,30,31,32,33,34,35,36,37,38]. The use of ceramic-ceramic or glass–ceramic composites is an effective way to tailor microstructure, electric, and thermal properties of functional materials for microwave substrates. In particular, this approach makes it possible to fabricate layered structures with buried passive electronic elements using advanced LTCC technology, which offers relatively low cost, flexibility in design and manufacturing, high miniaturization and integration degree.

Willemite Zn_2_SiO_4_ is a well-known ceramic material that crystallizes in a trigonal system, space group R-3. It exhibits excellent optical properties, high thermal conductivity, low thermal expansion coefficient, high thermal and chemical stability, and good mechanical properties. Willemite ceramics also show excellent microwave dielectric properties, namely a low dielectric constant (6–6.6), low dielectric loss (Qxf between 110,000 and 220,000 GHz) and a relatively low negative value of the temperature coefficient of resonance frequency (τ_f_ from −61 to −22 ppm/°C) [11,12,13]. To decrease the high sintering temperature of the willemite ceramics (above 1300 °C), various low melting glasses and sintering aids were added. However, publications devoted to the microwave applications of this material fabricated using LTCC technology are not numerous [23,24].

Zinc metaborate Zn_4_B_6_O_13_ has a cubic sodalite structure (space group I-43m) [25]. Its good photoluminescent and thermoluminescent properties are the basis for use in optical devices. A near-zero thermal expansion coefficient and high thermal conductivity (about 30 W/mK at 25 °C) are other advantageous features of this material [25]. However, Zn_4_B_6_O_13_ is much less popular for microwave applications in spite of its low dielectric constant of around 7 and low sintering temperature of around 950 °C [26].

The aim of this study is to develop a fabrication procedure and characterize Zn_4_B_6_O_13_-Zn_2_SiO_4_ composites from the point of view of their applicability as LTCC substrates for microwave and submillimeter wave circuits. Neither the fabrication of LTCC structures based on these materials nor their dielectric behavior at THz frequencies (predicted for 6G devices) have been explored previously.

## 2. Materials and Methods

For bulk ceramics, the fabrication procedure comprised solid-state synthesis of the components, milling of batches, uniaxial pressing of pellets, and sintering at 920–950 °C for 4 h. The starting components of the composites, Zn_4_B_6_O_13_ and Zn_2_SiO_4_ powders, were obtained via calcination at 900 °C and 1150 °C [23,26], respectively, followed by ball milling for 8 h (Pulverisette 5, Fritsch, Germany). The phase compositions of the powders after the synthesis and sintering processes were examined by the X-ray diffraction method, using Cu K_α1_ radiation within a 2Ɵ range of 5 to 90° (Empyrean, PANalytical, Almelo Netherlands The quantitative phase analysis and the crystal structure refinement were performed using the Rietveld method.

For the test LTCC substrates, the Zn_4_B_6_O_13_ and Zn_2_SiO_4_ powders were used to prepare slurries for tape casting. After ball milling for 8 h, the slurries containing the mixtures of the inorganic powders and polyvinyl butyral as a binder, fish oil as a dispersant, polyethylene glycol and dibutyl phthalate as plasticizers, and toluene and isopropyl alcohol as solvents, were cast using a tape caster (TTC-1200, Richard E. Mistler, Morrisville, PA, USA). The obtained green tapes (about 80 μm thick after drying) were cut into sheets, and the vias were fabricated by laser treatment (E-355-3-G-OA, Oxford Lasers, UK). The test conductive patterns were deposited, and the vias were filled using a precise screen printer (MT-320TVC, Micro-tec, Urayasu, Japan). The test structures consisted of 15 green sheets with the conductive patterns in the middle and on the top of a stack. Isostatic pressing was carried out using a laminator (IL-4008PC, Pacific Trinetics Corporation, Fremont, CA, USA) at 70 °C under a pressure of 20 MPa, 10 min.

Important information regarding shrinkage, softening, melting, and reflow temperatures for all composites as well as the composition of a eutectic was gathered on the basis of heating microscope studies (Leitz, Germany).

For selected green tapes, differential thermal analysis (DTA) was performed and thermogravimetric measurements (TG) were carried out using a thermal analyzer (STA 449 F3, Netzsch, Selb, Germany) in order to track the thermal effects and mass changes while heating at temperatures between 20 and 1000 °C and to optimize the firing profiles of the LTCC test structures.

The microstructure and elemental composition of the sintered substrates were analyzed using scanning electron microscopy (FEI Nova Nano SEM 200 with EDAX Genesis EDS system, Hillsboro, OR, USA) and X-ray energy dispersive spectroscopy (EDS). The quality of the screen-printed thick films and laminates was controlled using a Hirox digital microscope.

Dielectric properties were investigated at a frequency of 100 Hz–2 MHz and at temperatures between −30 and 150 °C using impedance spectroscopy (QuadTech 7600 LCR meter, Roslyn Heights, NY, USA) and at 0.1–3 THz at room temperature using time domain spectroscopy (TDS) (TPS Spectra 3000, Teraview, Cambridge, UK). In addition, for the 87% Zn_4_B_6_O_13_–13% Zn_2_SiO_4_ composite, the influence of the temperature changing in the 30–150 °C range was studied at THz frequencies. The THz measurements procedure has been described previously [23,26]. 

## 3. Results and Discussion

### 3.1. Composition, Microstructure, Sintering Behavior

**Figure 1:**
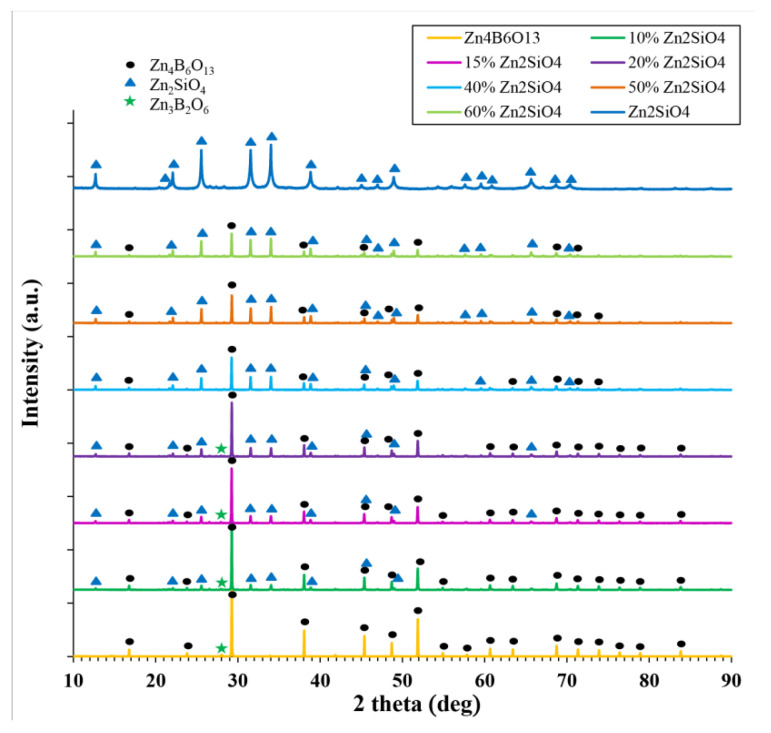
Diffraction patterns of Zn_4_B_6_O_13_–Zn_2_SiO_4_ composites sintered at 930–950 °C.

**Figure 2:**
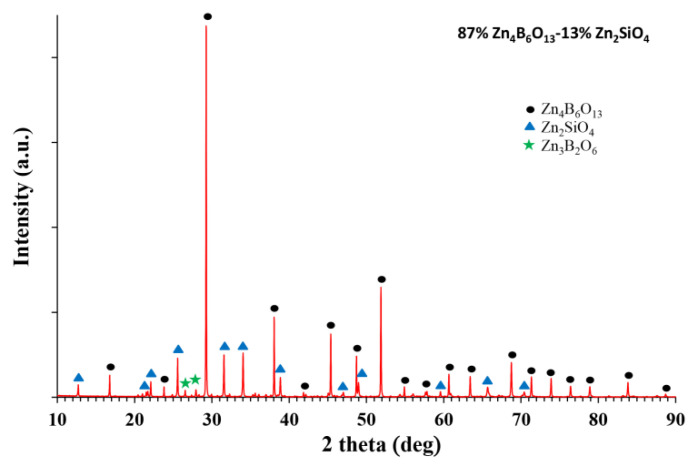
Diffraction pattern of 87% Zn_4_B_6_O_13_–13% Zn_2_SiO_4_ ceramic sintered at 930 °C.

Figure 1 shows a comparison of the XRD patterns of Zn_4_B_6_O_13_-Zn_2_SiO_4_ composites, pure willemite and pure zinc borate. Figure 2 illustrates a more detailed diffractogram of 87% Zn_4_B_6_O_13_–13% Zn_2_SiO_4_ ceramic.

Two dominant crystalline phases were detected—Zn_2_SiO_4_ (hexagonal crystallographic system, R-3 group) and Zn_4_B_6_O_13_ (cubic crystallographic system, I-43m group). For the samples containing 10, 13, 15, and 20% willemite, another zinc borate Zn_3_B_2_O_6_ was found as an additional crystalline phase. The amount of this phase was 7% for 87% Zn_4_B_6_O_13_–13% Zn_2_SiO_4_ ceramic and about 5% for the other materials from this group. For the ceramics with 40, 50, and 60% willemite, only two starting crystalline phases were found on the diffraction patterns. On the basis of data reported by other authors [39], in the ZnO–SiO_2_–B_2_O_3_ system, no reaction between zinc borate and zinc silicate should be expected.

The conditions necessary for a solid solution formation, which are well known, are a similar crystal structure, the same valence of the substituted ions and a less than 15% difference in the ionic radii. These conditions are not fulfilled for Zn_2_SiO_4_ and Zn_4_B_6_O_13_. These compounds crystallize in different crystallographic systems, although ZnO_4_ tetrahedra are common units for both structures. For B^3+^ and Si^4+^ being possible substituting ions, both their valences and ionic radii (0.12 and 0.26 Å, respectively for the coordination number of 4) are different. According to expectations, the results based on the Rietvield refinement gathered in Table 1 revealed only small changes in the lattice parameters of Zn_2_SiO_4_ and Zn_4_B_6_O_13_. Furthermore, no shift in the peak positions was observed in the diffraction patterns shown in Figure 1 and Figure 2. Thus, it was concluded that for Zn_4_B_6_O_13_–Zn_2_SiO_4_ ceramics, no solid solutions were formed.

**Figure 3:**
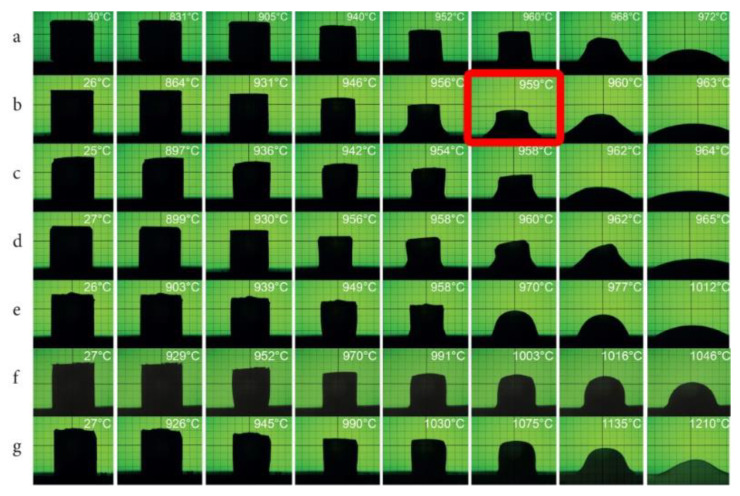
Selected images from a heating microscope for Zn_4_B_6_O_13_–Zn_2_SiO_4_composites: (**a**) 10% Zn_2_SiO_4_, (**b**) 13% Zn_2_SiO_4_ (in the red frame—melting point of the eutectic), (**c**) 15% Zn_2_SiO_4_ (**d**) 20% Zn_2_SiO_4_, (**e**) 40% Zn_2_SiO_4_, (**f**) 50% Zn_2_SiO_4_, (**g**) 60% Zn_2_SiO_4_.

Figure 3 shows selected images from a heating microscope for the composites being investigated.

It was found that the desired reduction in the sintering temperature below 960 °C was obtained with at least 40 wt % Zn_4_B_6_O_13_ in a Zn_4_B_6_O_13_–Zn_2_SiO_4_ composite. Another important conclusion from these studies is revealing of the formation of a eutectic with a melting point of 959 °C. The composition of this eutectic corresponds approximately to 87% Zn_4_B_6_O_13_–13% Zn_2_SiO_4_ composite. The oxide composition of this composite is 60.8% ZnO, 26.1% B_2_O_3_, and 13% SiO_2_ and is close to that of the eutectic revealed by Eidem et al. [39], which is 67 wt % ZnO, 19 wt % B_2_O_3_, and 14 wt % SiO_2_. As illustrated in Figure 3b, the shrinkage of the 87% Zn_4_B_6_O_13_–13% Zn_2_SiO_4_ composite begins at 864 °C, the softening temperature of this composite is 955 °C, and the melting point is 959 °C, which is a few degrees higher than the eutectic temperature of 950 °C indicated by Eidem et al. [39]. Very fast reflow occurs at 963°C. The heating behavior of 85% Zn_4_B_6_O_13_–15% Zn_2_SiO_4_ is almost the same as for 87% Zn_4_B_6_O_13_–13% Zn_2_SiO_4_, although the sample starts to shrink at a higher temperature of 897 °C. For the samples containing 13, 15, and 20% willemite, the formation of the hemisphere attributed to the melting point is about 960 °C, followed by rapid reflow at about 965 °C. For the samples with 10 and 40% Zn_2_SiO_4_, the melting and reflow are shifted by about 10–20 °C toward higher temperatures, and the sample reflow is not as rapid as for 87% Zn_4_B_6_O_13_–13% Zn_2_SiO_4_ and 85% Zn_4_B_6_O_13–_15% Zn_2_SiO_4_ composites. Meanwhile, the 50% Zn_4_B_6_O_13_–50% Zn_2_SiO_4_ and 40% Zn_4_B_6_O_13_–60% Zn_2_SiO_4_ composites exhibit distinctly higher melting temperatures of 1046 °C and 1135 °C, respectively.

**Figure 4:**
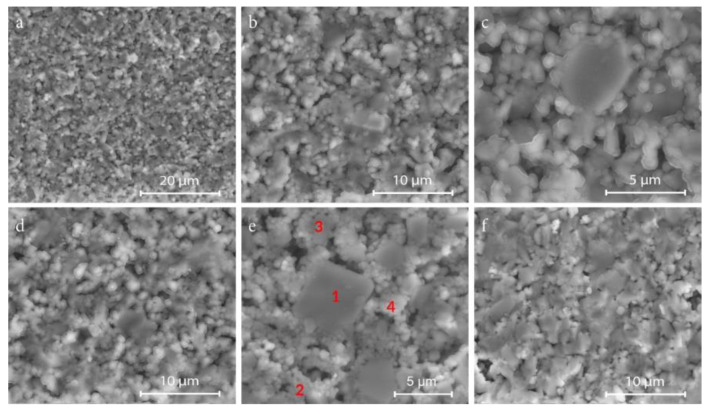
SEM images of fractured cross-sections of Zn_4_B_6_O_13_–Zn_2_SiO_4_ composites: (**a**), (**b**), (**c**) 13% Zn_2_SiO_4_, sintered at 930°C, ×5000, ×10,000, ×20,000, (**d**) 20% Zn_2_SiO_4_, ×10,000, sintered at 930 °C, (**e**) 50% Zn_2_SiO_4,_ sintered at 950 °C, ×20,000, marked points 1–4 of EDS analysis, (**f**) 60% Zn_2_SiO_4_, sintered at 950 °C, ×10,000.

SEM images in Figure 4 illustrate the microstructure of the Zn_4_B_6_O_13_–Zn_2_SiO_4_ composites. All ceramics are made of evenly distributed grains of zinc borate and zinc silicate and have a small closed porosity. The brighter areas can be attributed to Zn_2_SiO_4_ grains, for which the contribution of elements with higher atomic numbers (Si and a higher content of Zn) is greater than in the case of Zn_4_B_6_O_13_ (B and a lower content of Zn). Some borate grains are distinctly larger than those of the silicate (point 1 in Figure 4e), although small borate grains are also observed. The results of the EDS analysis presented in Table 2 at the points marked in Figure 4e indicate that point 1 corresponds to zinc borate grain, and point 4 can be attributed to zinc silicate grain. However, the EDS results are ambiguous due to the proportion of Zn, Si, and B atoms that differ significantly from the stoichiometric ratio (this can be explained by an imprecise detection of boron).

### 3.2. LTCC Structures

It was found that 40 wt % was the minimum Zn_4_B_6_O_13_ content that ensures that the sintering temperature is lowered to a level of 930–950 °C, which is acceptable for LTCC structures cofired with cheap commercial Ag-based conductive thick films. A higher willemite contribution caused an excessive increase in the sintering temperature. In previous studies [23,26], it was proved that both Zn_4_B_6_O_13_ and Zn_2_SiO_4_ ceramics do not react with silver, although the resolution, smoothness, and sharpness of the edges of the conductive patterns screen printed on the willemite-based substrates were better than those deposited on the zinc borate ceramics. Considering the requirements of a low sintering temperature suitable for cofiring with Ag-based commercial thick film pastes and the good quality of the printed conductors, the maximum preferred fraction of willemite in the Zn_4_B_6_O_13_- Zn_2_SiO_4_ composites is limited to 60 wt %.

**Figure 5:**
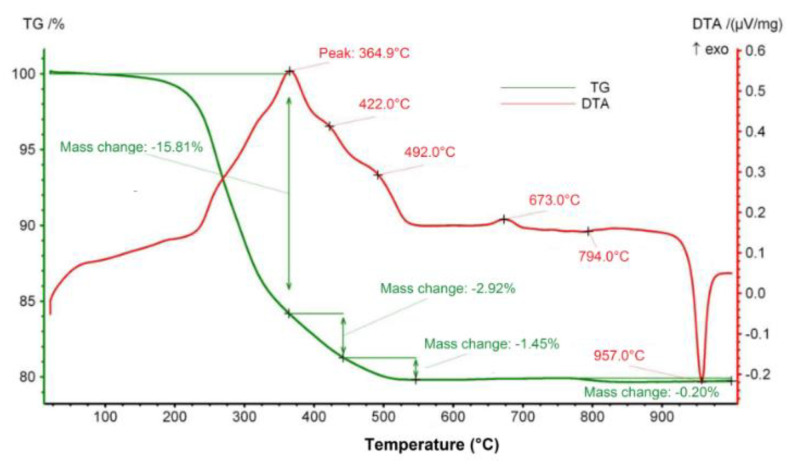
Thermogravimetric measurements (TG)–differential thermal analysis (DTA) of 50% Zn_4_B_6_O_13_–50% Zn_2_SiO_4_ green tape.

Figure 5 presents the results of the thermal analysis of a green ceramic tape based on 50% Zn_4_B_6_O_13_−50% Zn_2_SiO_4_ composite. The DTA curve shows a large exothermic peak in the temperature range 220–520 °C, which corresponds to a significant weight loss on the TG curve. This is the effect of burnout of the organic components of the green tape, mainly the binder, which is polyvinyl butyral.

A small exothermic peak at 673 °C, not accompanied by a change in mass, may be associated with the crystallization of another borate Zn_3_B_2_O_6_. A similar peak was observed at 654 °C by Ju et al. [28] and attributed to the formation of Zn(BO_2_)_2_ or Zn_3_B_2_O_6_. At about 790 °C, there is a very slight weight loss (0.2%), which may be associated with B_2_O_3_ evaporation. A distinct endothermic peak at 957 °C on the DTA curve corresponding to the unchanged sample weight is attributed to the melting of Zn_4_B_6_O_13_. A shift in the melting point to a lower temperature compared to pure Zn_4_B_6_O_13_ (980 °C) may indicate the influence of the second component of the composite (Zn_2_SiO_4_) and the formation of a eutectic between these phases. The location of the endothermic peak at 957 °C coincides very well with the melting point determined using a heating microscope for 87% Zn_4_B_6_O_13_–13% Zn_2_SiO_4_ sample (959 °C) and is close to the melting temperature of the eutectic reported by Eidem et al. [39] (950 °C).

**Figure 6:**
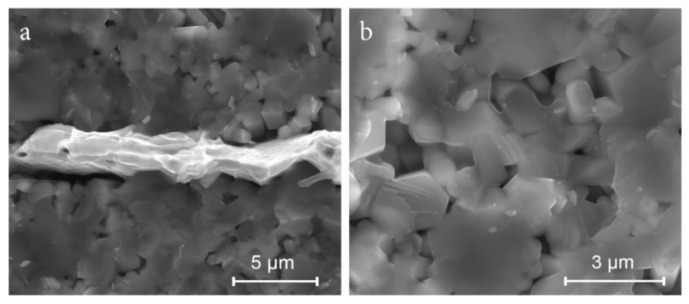
SEM of a low-temperature cofired ceramics (LTCC) structure based on 50% Zn_4_B_6_O_13_–50% Zn_2_SiO_4_ ceramic sintered at 950 °C (**a**) ceramic layers with internal screen-printed AgPd thick film, (**b**) ceramic layer.

Figure 6 illustrates the dense microstructure of a layered structure based on Zn_4_B_6_O_13_–Zn_2_SiO_4_ ceramic co-sintered at 940 °C with AgPd-based electrodes. The porosity of the ceramic layers is low, and the cooperation with the metallic layer is very good.

### 3.3. Dielectric Properties

**Figure 7:**
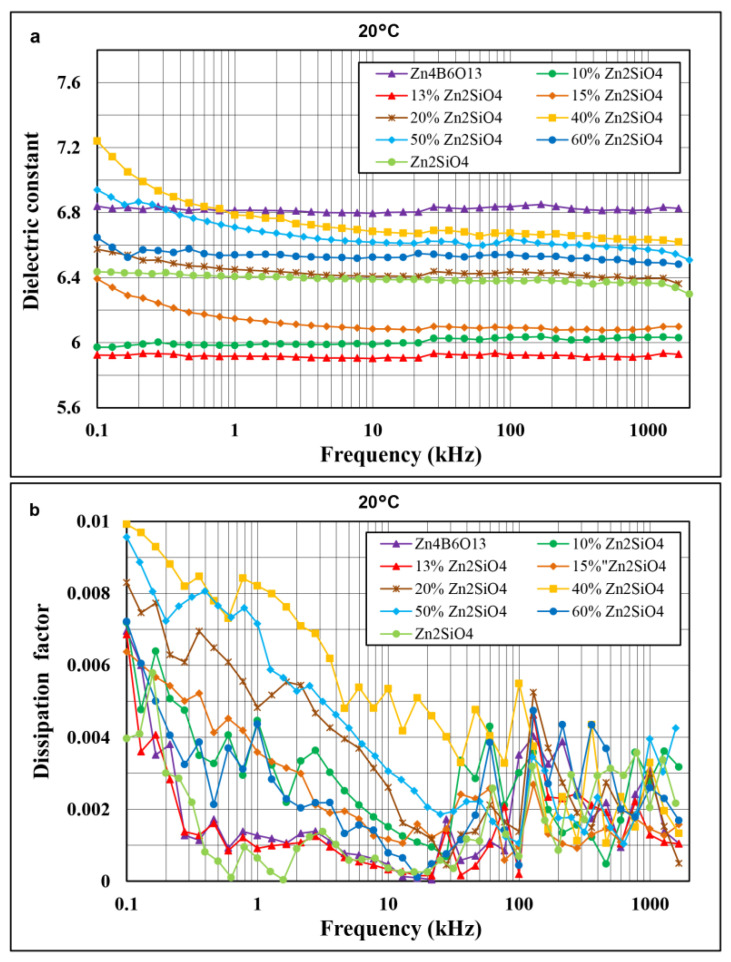
Comparison of frequency dependences of dielectric constant (**a**) and dissipation factor (**b**) at 20 °C in the range of 100 Hz-2 MHz for Zn_4_B_6_O_13_, Zn_2_SiO_4_, and Zn_4_B6O_13_–Zn_2_SiO_4_ composites.

In Figure 7a,b, the frequency dependences of the dielectric constant and dissipation factor at 20 °C in the range of 100 Hz to 2 MHz are compared for pure Zn_4_B_6_O_13_, pure Zn_2_SiO_4_, and seven Zn_4_B_6_O_13_–Zn_2_SiO_4_ composites.

These ceramics were sintered at optimal sintering temperatures, which were similar in the case of the composite materials and Zn_4_B_6_O_13_ (930–950 °C) and significantly different in the case of pure Zn_2_SiO_4_ ceramic (1320 °C). The differences in the sintering temperature and the level of closed porosity should be taken into account as an additional factor influencing the dielectric properties. The dielectric constant values of the investigated materials are low, ranging from 5.9 to 6.9. The dissipation factor values are below 0.006 above 3 kHz. Relatively strong Zn–O, B–O, and Si–O covalent bonds and a strong hybridization of Zn 3d and O 2p orbitals promote low dielectric constants and low dielectric losses of the composites. Among these materials, the highest dielectric constant of 6.8 at 1 MHz was found for pure Zn_4_B_6_O_13_ ceramics sintered at 940 °C, the lowest of 5.9 at 1 MHz was found for 87% Zn_4_B_6_O_13_–13% Zn_2_SiO_4_ sintered at 930 °C. The lowest dissipation factor values exhibit the materials with the highest densification degree—pure willemite, pure zinc metaborate, and the composites with 10, 13, and 15% Zn_2_SiO_4_. For all materials under investigation, the frequency dependences of the dielectric constant are weak above 1 kHz. An increase in the dielectric constant and dissipation factor at low frequencies (below 3 kHz) is attributed to contribution of Maxwell–Wagner polarization effects related to the presence of porosity, secondary phases, grain boundaries, and space charge. At low frequencies, more distinct differences between the dissipation factors of the composites were observed due to the dependence of extrinsic losses on a level of porosity, glassy phase fraction, and grain sizes.

**Figure 8:**
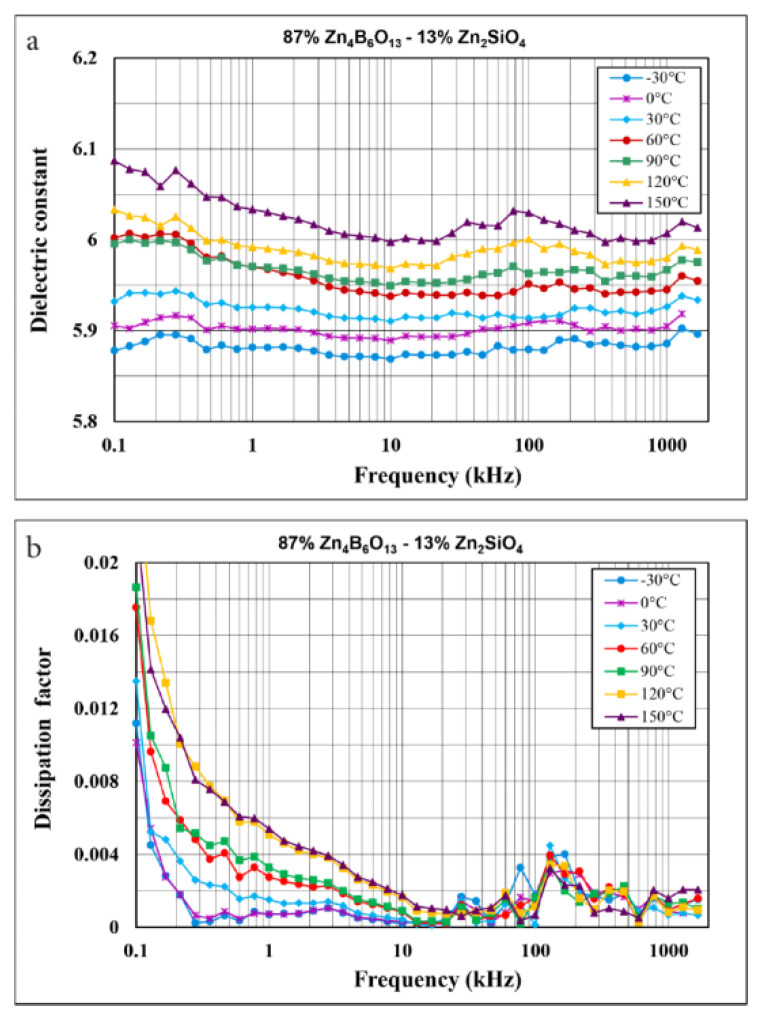
Dielectric constant (**a**) and dissipation factor (**b**) as a function of frequency in the range 100 Hz to 2 MHz and in the temperature range –30 to 150 °C for 87% Zn_4_B_6_O_13_–13% Zn_2_SiO_4_ sintered at 930 °C.

Figure 8a illustrates the changes in the dielectric constant of 87% Zn_4_B_6_O_13_–13% Zn_2_SiO_4_ as a function of frequency in the 100 Hz to 2 MHz range and for a few temperatures between −30 and 150 °C.

This ceramic shows low dielectric constant values of 5.9–6.1 within these frequency and temperature ranges, slightly increasing with temperature and weakly dependent on frequency. The dissipation factor is rather low (0.0003–0.004) above 3 kHz (Figure 8b). A higher dissipation factor observed for the lowest frequencies tested and the highest temperatures can be attributed to the losses related to space charge polarization.

**Figure 9:**
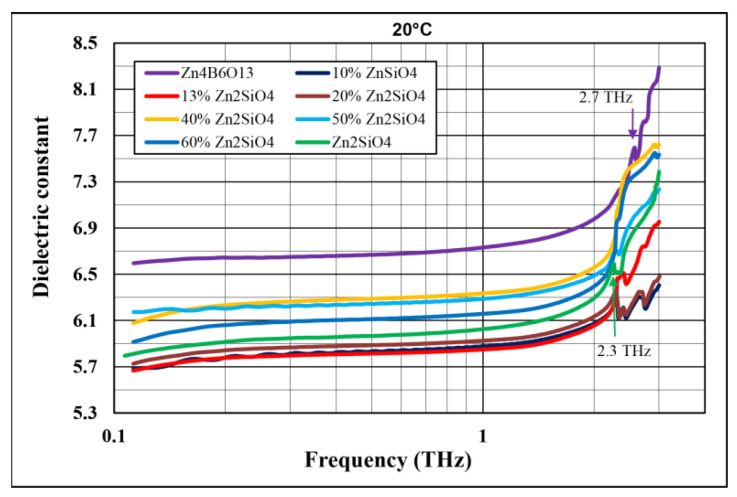
Comparison of dielectric constant at 20 °C as a function of frequency in the range 0.12–3 THz for Zn_4_B_6_O_13_, Zn_2_SiO_4_, and Zn_4_B_6_O13–Zn_2_SiO_4_ ceramics.

Figure 9 shows a comparison of the dielectric constant at room temperature in the 0.1–3 THz frequency range for pure Zn_4_B_6_O_13_, pure Zn_2_SiO_4_, and Zn_4_B_6_O_13_–Zn_2_SiO_4_ composites. The plots of the dielectric constant versus frequency have similar courses for all the materials investigated. In the frequency range of 0.1–1.5 THz, no peaks are present, and the frequency dependence is weak. The dielectric constants at 1 THz are slightly lower than those at 1 MHz. In general, such frequency dependences of the dielectric constant at very high frequencies are consistent with the theoretical predictions of the model of damped harmonic oscillators [40,41].

Above 2 THz, a rapid increase in dielectric constant and a few characteristic local maxima were observed. Similarly to the range of 100 Hz to 2 MHz, the lowest dielectric constants, assuming values of 5.84 and 5.87 at 1 THz, were found for 87% Zn_4_B_6_O_13_–13% Zn_2_SiO_4_, and 90% Zn_4_B_6_O_13_–10% Zn_2_SiO_4_, respectively. A distinct local maximum or an inflection point of the dielectric constant versus frequency curve was revealed at about 2.3 THz for Zn_2_SiO_4_ and all the composites examined. The presence of a strong absorption peak at the same frequency was revealed for willemite ceramics by Nedelcu et al. [17] in the THz-TDS measurements and attributed to the lowest-frequency phonon mode. These authors also stated that below 2 THz, zinc silicate ceramics exhibit a low absorption coefficient value, which is consistent with the results of this study. The presence of a secondary phase Zn_3_B_2_O_6_ (revealed by XRD analysis) does not affect the dielectric constant of the composites containing this phase due to its small amount (a few percent) and the very close values of the dielectric constant of both zinc borates (6.7 at 11 GHz for Zn_3_B_2_O_6_ [29]).

**Figure 10:**
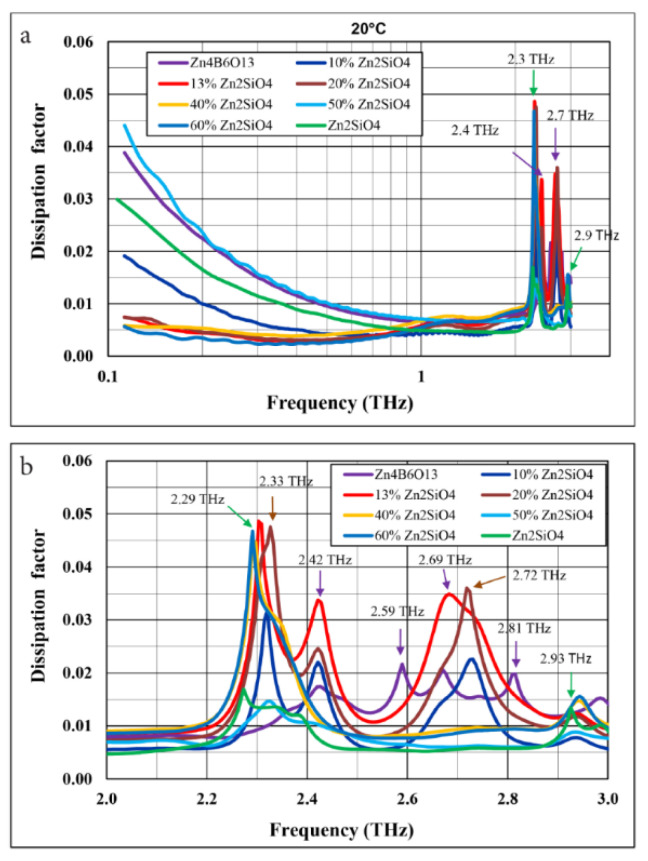
Comparison of dissipation factor at 20 °C as a function of frequency for Zn_4_B_6_O_13_, Zn_2_SiO_4_, and Zn_4_B_6_O_13_–Zn_2_SiO_4_ ceramics: (**a**) a log-lin plot in the range 0.12–3 THz and (**b**) a more detailed lin-lin plot, in the range 2–3 THz.

For the frequency dependence of the dissipation factor (Figure 10a), a local minimum and a plateau in the frequency range of 0.6–1.6 THz is generally observed for all materials. At 1 THz, the dissipation factors change from 0.004 to 0.006. The lowest dielectric loss was displayed by pure willemite and the 90% Zn_4_B_6_O_13_–10% Zn_2_SiO_4_ composite. The peaks observed in the dissipation factor versus frequency plots are more distinct and slightly shifted toward lower frequencies as compared with the maxima on the dielectric constant plots. It can be seen from the more detailed plot (Figure 10b) that the dissipation factor peaks for pure willemite occur at 2.3 and 2.9 THz, while for Zn_4_B_6_O_13_ ceramic, they occur at 2.4, 2.6, 2.7, and 2.8 THz. For all the composites, the dissipation factor peaks observed at 2.3 and at 2.9 THz can be attributed to willemite. The assignment of other peaks is more ambiguous, although the peaks observed for Zn_4_B_6_O_13_-rich compositions at about 2.4 THz and at about 2.7 THz can be related to the predominant borate component.

**Figure 11:**
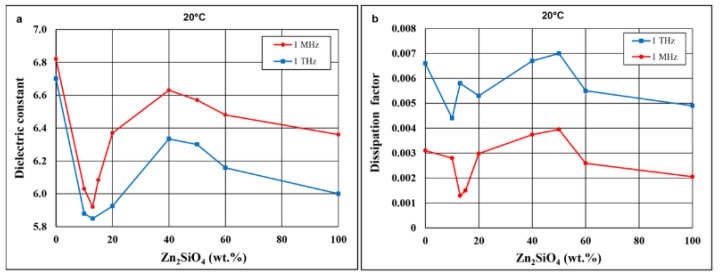
Dependence of dielectric constant (**a**) and dissipation factor (**b**) at 20 °C on the Zn_2_SiO_4_ content in Zn_4_B_6_O_13_–Zn_2_SiO_4_ composites for 1 MHz and 1 THz.

As illustrated in Figure 11a, the dielectric constant does not decrease linearly with the willemite content, which is a composite component with a lower dielectric constant.

An interesting effect of the distinct minimum of the dielectric constant occurs for compositions with 10–15% Zn_2_SiO_4_. The corresponding SiO_2_ content in these composites is close to the composition of the eutectic revealed by Eidem et al. [39] and observed in this study during heating microscope studies. The shape of the plots of both the dielectric constant and the dissipation factor versus the willemite content (Figure 11a,b) are similar at 1 MHz and 1 THz. It follows from Figure 11b that the dissipation factors of the materials under investigation, which were fired each at its optimal sintering temperature, do not differ significantly both at 1 MHz and 1 THz.

**Figure 12:**
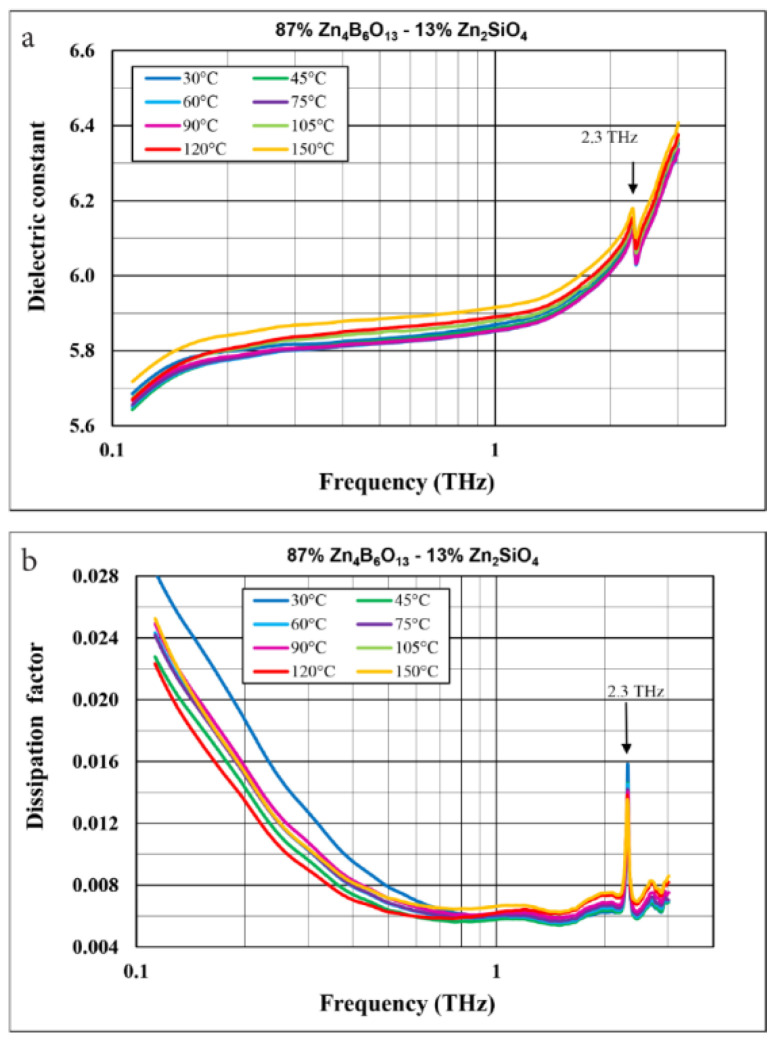
Dielectric constant (**a**) and dissipation factor (**b**) as a function of frequency in the range 0.12–3 THz and in the temperature range −30 to 150 °C for 87% Zn_4_B_6_O_13_–13% Zn_2_SiO_4_ ceramic sintered at 930 °C.

**Figure 13:**
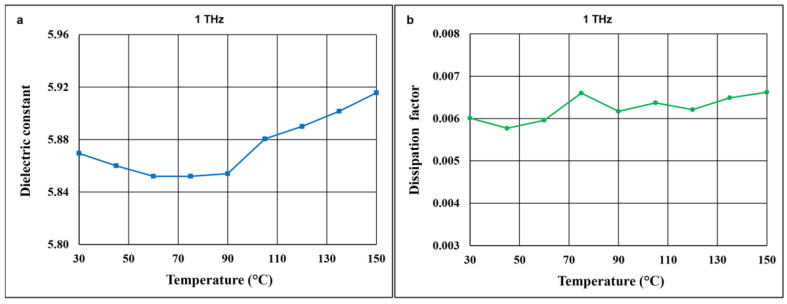
Dielectric constant (**a**) and dissipation factor (**b**) at 1 THz versus temperature in the 30–150 °C range for 87% Zn_4_B_6_O_13_–13% Zn_2_SiO_4_ ceramic sintered at 930 °C.

Since the temperature coefficient of thermal expansion is very low for both willemite and Zn_4_B_6_O_13_, the main contribution to the temperature coefficient of resonant frequency for the composites under investigation originates from the temperature coefficient of dielectric permittivity. Figure 12 shows the effect of temperature on the dielectric constant and the dielectric loss in the terahertz range for 87% Zn_4_B_6_O_13_–13% Zn_2_SiO_4_ ceramic. The course of the frequency dependences is the same for different temperatures in the tested range of 30–150 °C. As illustrated in Figure 13a,b, for frequencies corresponding to stable characteristics, the dielectric constant and the dissipation factor values generally slightly increase with temperature. These changes are small, less than 10% in the temperature range of 30–150 °C for the dielectric constant. The temperature coefficient of dielectric permittivity in the 30–90 °C temperature range is relatively low, at a level of −44 ppm/°C for 87% Zn_4_B_6_O_13_–13% Zn_2_SiO_4_ ceramic.

For single phase and well densified ceramics, extrinsic dielectric losses associated with porosity, grain boundaries, secondary phases, crystal defects, etc. should be negligible at microwave and mm-wave frequencies [40,41]. The dominant role is supposed to be played by intrinsic losses related to the crystal lattice vibrations determined by the crystal structure characteristics [42,43,44]. Ionic polarizability, packing fraction, bond valence, and bond strength are crucial factors that enable the theoretical prediction of high-frequency dielectric properties. A higher packing fraction entails less space for lattice vibration, which leads to a lowering of intrinsic losses [42]. A high bond valence is also an important factor that decreases the dielectric constant, the dielectric loss, and the absolute value of the temperature coefficient of resonant frequency [43,44]. The high bond valence decreases intrinsic dielectric losses due to a weaker damping of the signal by the reduction of anharmonic phonon interactions [43].

The linear increase in the imaginary part of dielectric permittivity (proportional to the dissipation factor) predicted according to the model of damped harmonic oscillators [40] was not observed in the THz range for the composite materials under investigation. The lowest deviation from this behavior was revealed for composites containing 10, 13, 20, and 40% Zn_2_SiO_4_, which exhibit the highest densification degree. A general reason for the discrepancy between the predicted and observed dielectric losses may be the presence of numerous interphases in the composites built of two materials, and additionally for compositions with 10–20% Zn_2_SiO_4_, the presence of a secondary phase Zn_3_B_2_O_6_, which leads to a marked contribution of extrinsic losses, even at very high frequencies.

## 4. Conclusions

Low dielectric constant substrates for microwave and submillimeter wave applications in the form of bulk ceramics and LTCC multilayer structures based on Zn_4_B_6_O_13_–Zn_2_SiO_4_ composites were successfully prepared in processes comprising solid-state synthesis, milling, tape casting, screen printing, isostatic lamination, and sintering.

The merits of using the Zn_4_B_6_O_13_–Zn_2_SiO_4_ composites instead of ceramics based on pure willemite or pure zinc metaborate are the following:

- Lowering or broadening and stabilization of the sintering temperature range
- Improvement of the surface smoothness and uniformity of the substrates and consequently the quality and resolution of screen-printed patterns
- High-frequency dielectric constants for composites containing 10–15 wt% Zn_2_SiO_4_ are lower than those of the pure ceramic components

The most advantageous properties were shown by 87% Zn_4_B_6_O_13_–13% Zn_2_SiO_4_ composite, namely a low sintering temperature of 930 °C, good compatibility with Ag and Ag-Pd based commercial thick film pastes, a low dielectric constant of about 5.8 in the 0.15-1.1 THz range, a relatively low dissipation factor of 0.006 at 1 THz, and a relatively low absolute value of the temperature coefficient of dielectric permittivity of –44 ppm/°C.

## Figures and Tables

**Table 1:** Lattice parameters of Zn_4_B_6_O_13_–Zn_2_SiO_4_ composites.

Lattice Parameters	Zn_2_SiO_4_ Content (wt %)					
	10	13	15	20	40	60
“a” Zn_4_B_6_O_13_ (Å)	7.4746	7.4749	7.4749	7.4748	7.4745	7.4743
“a” Zn_2_SiO_4_ (Å)	13.9326	13.9352	13.9350	13.9351	13.9303	13.9324
“c” Zn_2_SiO_4_ (Å)	9.3066	9.3066	9.3067	9.3081	9.3043	9.3060

**Table 2:** Results of EDS analysis at the points marked in Figure 4e for 50% Zn_4_B_6_O_13_–50% Zn_2_SiO_4_ ceramic sintered at 950 °C.

Element	at %			
	Point 1	Point 2	Point 3	Point 4
B	50.66	38.37	30.19	0.00
O	27.15	39.06	29.05	37.03
Si	1.05	3.72	5.32	19.11
Zn	21.15	18.85	35.44	43.86

## Data Availability

The data presented in this study are available on request from the corresponding author. The data are not publicly available as the data also form part of an ongoing study.
